# Doctors' Wills

**Published:** 1914-03-28

**Authors:** 


### Doctors' Wills.

Mr. Bertram Thornton, M.R.C.S., L.R.C.P., of Margate, consulting surgeon to the Royal Sea Bathing Hospital, has left estate of the gross value of £12,334, of which £10,694 is net personalty.

Mr. Samuel Aaron Welch, M.R.C.S., of Newcastle-on-Tyne, left estate valued at £25,078 gross, with net personalty £3,127. He requested his trustees to retain (among other investments) his Kent Colliery shares for a period of ten years.

Dr. Frank Buszard, M.D., F.R.C.P., F.R.C.S., for over fifty years hon. physician to the Northampton Hospital, one of the pioneers of hospital reform, and for some years leader of the Unionist Party in the district, has left estate of the gross value of £13,374, of which £10,161 is net personalty.

